# Anti-glycine receptor antibody-positive progressive encephalomyelitis with rigidity and myoclonus initially presenting with one-sided stiff face: A case report

**DOI:** 10.3389/fneur.2022.1021437

**Published:** 2022-10-26

**Authors:** Ken-Ichi Irie, Takahisa Tateishi, Taiga Moritaka, Naonori Sakurada, Shinsuke Kikuchi, Takayuki Taniwaki

**Affiliations:** Division of Respirology, Neurology and Rheumatology, Department of Medicine, Kurume University School of Medicine, Kurume, Japan

**Keywords:** PERM, anti-GlyR antibody, stiff-person syndrome, myoclonus, rigidity, hyperekplexia

## Abstract

**Background:**

Progressive encephalomyelitis with rigidity and myoclonus (PERM) is a subtype of stiff-person syndrome, a rare cerebrospinal disease that causes brainstem symptoms, myoclonus, muscle rigidity, and hyperekplexia.

**Case presentation:**

A 71-year-old man experienced left-sided stiff face, and was subsequently admitted to our hospital because of the appearance of left-dominant lower limb myoclonus. Muscle rigidity followed 3 days later. Magnetic resonance imaging revealed no abnormality. An electrophysiological examination showed a toughness of the antagonistic muscle following evocation of the Achilles tendon reflex, and a tonic phenomenon affecting the left facial muscles during the blink reflex. The patient's serum was positive for anti-glycine receptor (anti-GlyR) antibody, suggesting PERM. The patient was administered steroids, immunoglobulin therapy, and immunosuppressive drugs. He gradually improved after these therapies and became able to walk using a walker.

**Conclusions:**

We conclude that this was a rare case of anti-GlyR antibody-positive PERM with unilateral brainstem symptoms, myoclonus, and muscle rigidity.

## Introduction

Progressive encephalomyelitis with rigidity and myoclonus (PERM) is a subtype of stiff-person syndrome consisting of limb/trunk muscle rigidity, hyperekplexia, and myoclonus (1). Anti-glycine receptor (anti-GlyR) antibody has been implicated in its pathogenesis (2). In some anti-GlyR antibody-positive PERM cases reported to date, the initial symptoms were stiffness of the lower limbs and myoclonus. In others, brainstem symptoms preceded disease (3). Here, we encountered a case of PERM which initially manifested with trismus due to one-sided stiff face.

## Case presentation

A 71-year-old man whose past medical history was unremarkable experienced sudden trismus and left facial stiffness was admitted to the hospital. On the third day of the patient's hospitalization, left-dominant muscle rigidity, pain, and intermittent myoclonus appeared in both lower limbs, and he was transferred to our hospital. A neurological examination on admission revealed decreased pain and temperature sensation in the mandibular nerve area and muscle rigidity of the left face, in addition to trismus, hoarseness, muscle rigidity of the left lower limb, myoclonus, and hyperekplexia with left lower limb predominance. He had no sensory impairment and had enhanced deep tendon reflexes in his extremities, but no pathological reflexes. One day prior to transfer, dysautonomia appeared, including dysuria, constipation, and excessive sweating of the trunk and extremities. Laboratory tests, including hematological and biochemical analyses, revealed that levels of autoantibodies (anti-nuclear antibodies, anti-SS-A antibody, anti-SS-B antibody), hepatitis B virus antigen, anti-hepatitis C virus antibody, anti- human T-cell leukemia virus type I/II antibodies and tumor marker (carcinoembryonic antigen, colorectal carcinoma antigen 19–9), were normal. However, his erythrocyte sedimentation rate was elevated at 58 mm/h, as was his IgG level at 1981 mg/dl.

**Figure 1:**
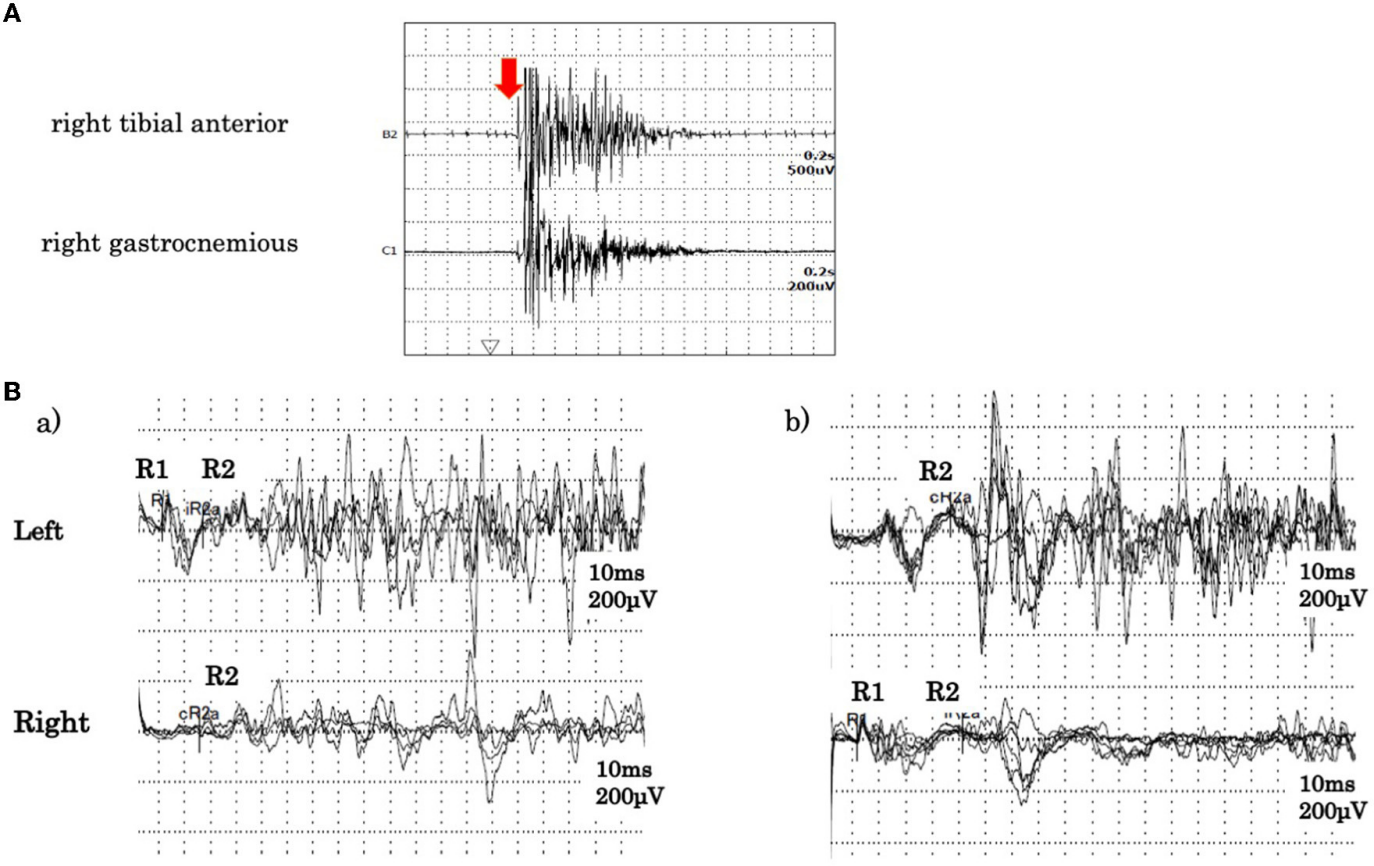
**(A)** Surface electromyogram findings. During the Achilles tendon reflex, tibialis anterior and gastrocnemius muscles exhibited continuous simultaneous co-contraction. The arrow indicates tapping of the Achilles tendon. **(B)** Blink reflex findings. (a) Record obtained on stimulation of the left side. (b) Record obtained on stimulation of the right side. In both (a,b), higher amplitude responses of R1 and R2 were observed from the left eyelid.

**Figure 2:**
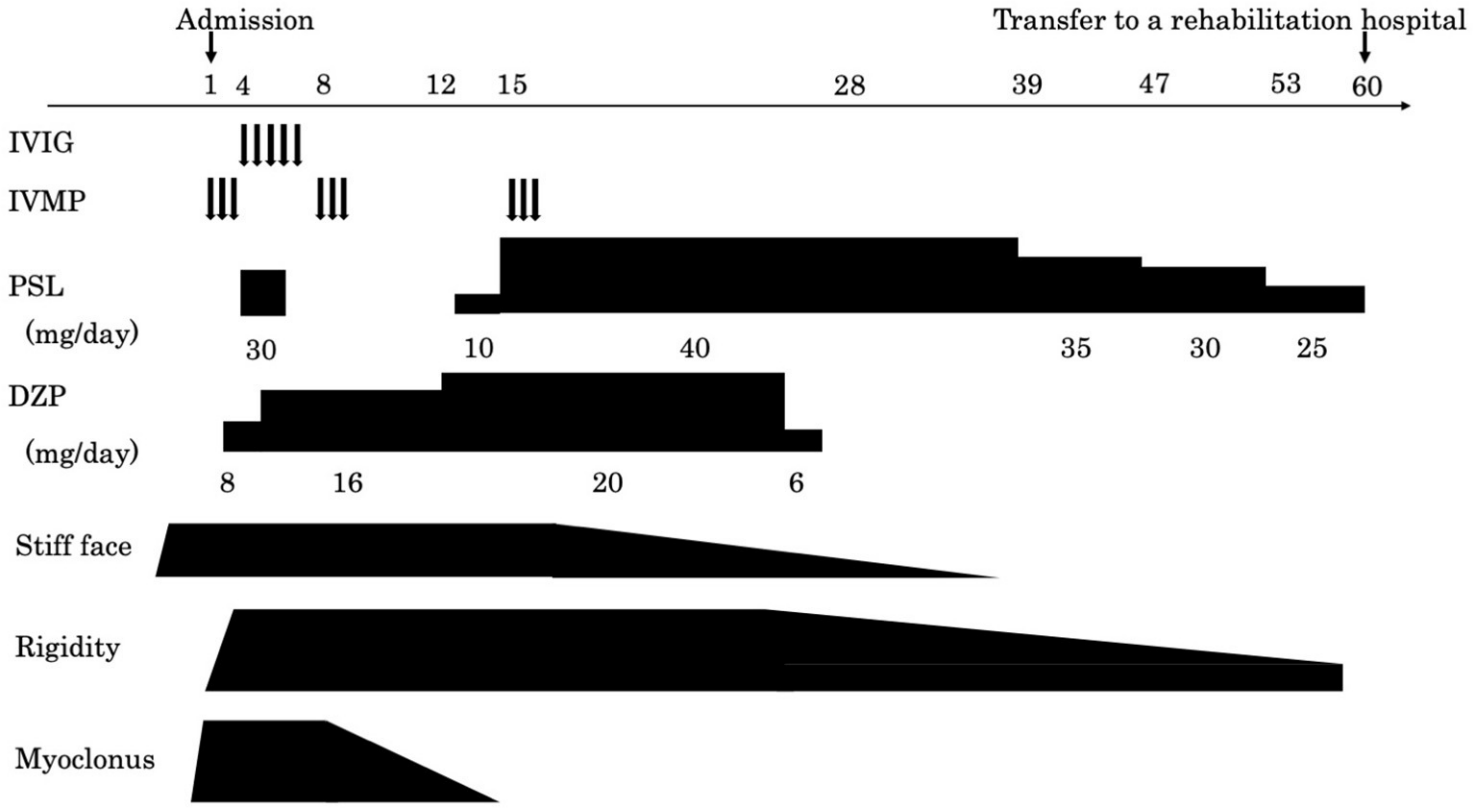
A graphical summary of symptom onset and clinical management for the current case. The arrow on the left indicates the date of admission and the arrow on the right indicates the date of transfer.

A cerebrospinal fluid analysis showed a normal cell count of 3/μL (100% mononuclear cells) and a normal protein level (30 mg/dL). No oligoclonal band was detected. We tested for the presence of the following serum anti-neuronal antibodies: amphiphysin, CV2/collapsin response mediator protein 5, Ma2/Ta, Ri, Yo, Hu, Recoverin, SRY-Box transcription factor 1, titin, Zic, glutamic acid decarboxylase65, Tr, and GlyR. Of these, only anti-GlyR antibody yielded positive reactions in serum and cerebrospinal fluid. Surface electromyogram of the left tibialis anterior and left gastrocnemius muscles revealed continuous, simultaneous co-contraction of both muscles on tapping the Achilles tendon with a reflex hammer. This is suggestive of rigidity in both the agonist and antagonist muscles of the Achilles tendon reflex (Figure 1A). Both the R1 and R2 components of the blink reflex showed higher amplitude on the left compared with the right after stimulation of either side, indicating hyper-excitability on the left side (Figure 1B). Head magnetic resonance imaging showed no structural abnormalities. A whole body computed tomography showed a gastric submucosal tumor, which was diagnosed as a hyperplastic polyp by gastrointestinal endoscopy. We administered three courses of steroid pulse therapy with methylprednisolone (1,000 mg/day; duration 3 days) on the 1st, 8th, and 15th day after admission, as well as high-dose immunoglobulin (400 mg/kg/day; duration 5 days) on the 4th day. Following the third administration of steroid pulse therapy, we administered prednisolone (40 mg/day) and tapered. As symptomatic treatment, the patient was administered diazepam (20 mg/day, oral) and dantrium (75 mg/day, oral). After those steroid therapies, the muscle rigidity of the whole body and the myoclonus of both lower limbs gradually improved. The patient was then transferred for rehabilitation on the 60th day after admission. By the 90th day, his score on the modified Rankin Scale had improved from 5 to 3 (Figure 2).

## Discussion and conclusions

Brown et al. first described PERM as a subacute disseminated encephalomyelitis with muscle rigidity and myoclonus, which has a distinct course and symptoms from classical stiff-person syndrome (1). The antibodies involved in PERM include anti-GAD antibody, amphiphysin antibody, anti-gephyrin antibody, and anti-GlyR antibody, all of which are involved in the inhibitory neurotransmission (4). In this case, we confirmed that the antibody was negative for amphiphysin and GAD antibodies, the cause of stiff person syndrome. Although leucine-rich glioma-inactivated 1 and contactin-associated protein-like 2 was not measured, the absence of fasiobrachial dystonic seizure, myotonia, and myokymia ruled out anti-voltage-gated potassium channels complex antibody-related disease. Ultimately, we diagnosed PERM based on positive anti-GlyR antibody in serum and cerebrospinal fluid. The glycine receptor is a pentameric ligand-gated chloride channel that is distributed throughout the brainstem and spinal cord. When glycine binds to these ionotropic receptors on a cell's surface, chloride ions flow into the cell, suppressing post-synaptic membrane excitation (5).

In the brainstem, the GlyR is expressed in the principal sensory nucleus and motor nuclei of the trigeminal nerve, the facial motor nucleus, and the dorsal motor nucleus of the vagus nerve. It is also found on the anterior and posterior horn cells of the spinal cord. While the mechanism of GlyR autoantibody action is not clear, suppression of GlyR function is assumed to induce excessive responses to sensory inputs, such as those from muscle spindles, somatosensory stimuli, and light and sound stimuli. As a result, these stimuli trigger symptoms including facial rigidity, hoarseness, myoclonus, and hyperekplexia of both lower limbs. Stiff-limb syndrome is characterized by the localized involvement of one limb, usually a leg. Unilateral cerebral and spinal cord lesions have been reported on MRI scans in these patients (3). This case presented with symptoms of unilateral brainstem dysfunction, which were followed by symptoms affecting the lower limb on the same side. This suggests that both spinal cord and brainstem lesions are confined to one side. In the electrophysiological examination, the amplitude of both the R1 and R2 components of the blink reflex was higher on the left side after stimulation of either side, and we could visually capture the moment at which the α motor neuron was released from suppression. The R1 response is mediated through the ipsilateral principal sensory nucleus of the trigeminal nerve, while the R2 response is mediated through multiple pathways, including the spinal trigeminal nucleus and the bilateral lateral reticular formation *via* the trigeminal nucleus sensory nucleus. In the present case, both R1 and R2 showed high amplitude on the left side, suggesting hyperexcitability in the facial nucleus and the motor nucleus of the trigeminal nerve as opposed to the internuclear pathway. Additionally, the surface electromyograms of the left tibialis anterior and the left gastrocnemius muscles showed co-contraction and tonicity of both muscles during the Achilles tendon reflex. This indicates excessive excitability of the anterior horn cells of the spinal cord. Iizuka et al. reported that trismus was due to muscle stiffness induced by continuous excitation of alpha motor neurons in the brainstem (5). A summary of published seropositive GlyR antibody-positive PERM cases (including the current case) is provided in Table 1. In recent case reports, brainstem symptoms appeared initially, while myoclonus and rigidity of the lower limbs, hyperekplexia, and painful spasms appeared later. Therefore, there is often a delay between the onset of disease and subsequent diagnosis and treatment. In a case series of 45 glycine receptor antibody-positive PERM cases, most patients showed marked improvement with immunotherapies (17). As introducing immunotherapy within 2 months of onset has been reported to improve prognosis (6), early diagnosis is of great importance. In the present case, we administered immunotherapies from a relatively early stage, sparing the patient from ventilator usage and improving his symptoms promptly.

**Table 1:** A summary of published seropositive GlyR antibody positive PERM cases (including the current case).

**References**	**Age of onset, sex**	**Days from onset to immunotherapy**	**Symptom of onset**	**Immunotherapy**	**Respilatory disturbance**	**mRS after immunotherapy**	**Outcome**
Chang et al. (6)	46, M	1 months	Brain stem syndrome	IVIG, IVMP, PE Oral corticosteroid, AZP	+	5	No recurrence
Yao et al. (7)	52, M	45 days	Headache, dysarthria	IVMP, IVIG oral corticosteroid	+	1	No recurrence
Bernard et al. (8)	78, M	3 months	Seizure, cognitive imparement	IVMP, IVIG PE, AZP	-	4	He died of respiratory infection after 2.5 years
Fujino et al. (9)	62, M	7 weeks	Painful spasm in a leg	IVMP, IVIG Oral corticosteroid	+	1	No recurrence
Mizutani et al. (10)	59, M	2 weeks	Brain stem syndrome	IVMP, IVIG Dxazosin, Cyclosporine RTX, Cyclophosphamide	+	5	He remained bedridden with a tube feeding
Wang et al. (11)	61, M	11 days	Left facial weakness	IVMP, IVIG Oral corticosteroid, mycophenolate mofetil	-	5	No recurrence
Shimazaki et al. (12)	63, M	7 months	Painful stiffness in legs, dysphagia	IVMP, IVIG Oral corticosteroid	-	4	No recurrence
Borellini et al. (13)	60, M	3 months	Painful spasms of trunk and legs	IVMP, IVIG Oral corticosteroid	-	5	He improved after chemotherapy for Hodgkin's lymphoma
Ozaki et al. (14)	75, F	None noted	Rigidity of legs	PE	-	5	He died of respiratory failure after 10 months
Lee et al. (15)	41, F	2 weeks	Hypersomnia, dysphagia, gait disturbance	IVMP, IVIG Oral azathioprine and corticosteroid	-	Not noted	No recurrence
Gluck et al. (16)	63, M	5 months	Sleep requirements and disorganized behavior	High dose steroids, PE, rituximab	+	5	He remained bedridden with a tracheostomy. Modified Rankin scale score is 4 6 months after discharge.
Current case	71, M	3 days	Left stiff face	IVMP, IVIG Oral corticosteroid	-	5	No recurrence

In conclusion, this is a rare case which presented with hemifacial stiffness (a symptom of unilateral brainstem dysfunction), before developing rigidity and myoclonus with dominance on the left side. Our ability to assess face stiffness and stiffness of the lower limbs electrophysiologically, thereby demonstrating the cause of these symptoms objectively, was key to this diagnosis. Despite the rare initial presentations of this case, the clinical outcome was positive due to early diagnosis and treatment.

## Data availability statement

The original contributions presented in the study are included in the article/supplementary material, further inquiries can be directed to the corresponding author.

## Ethics statement

Ethical review and approval was not required for the study on human participants in accordance with the local legislation and institutional requirements. The patients/participants provided their written informed consent to participate in this study.

## Author contributions

K-II, TTat, and TTan made the study plan, conducted the research, and wrote the paper. TM, NS, and SK collected patient's samples and clinical data and supervised the analyses. All authors contributed to the article and approved the submitted version.

## Funding

This work was partly supported by Cognitive and Molecular Research Institute of Brain Diseases, Kurume University School of Medicine.

## Conflict of interest

The authors declare that the research was conducted in the absence of any commercial or financial relationships that could be construed as a potential conflict of interest.

## Publisher's note

All claims expressed in this article are solely those of the authors and do not necessarily represent those of their affiliated organizations, or those of the publisher, the editors and the reviewers. Any product that may be evaluated in this article, or claim that may be made by its manufacturer, is not guaranteed or endorsed by the publisher.

## Abbreviations

anti-GlyR: anti-glycine receptor
PERM: progressive encephalomyelitis with rigidity and myoclonus.
